# Microbial Alpha-Amylase Production: Progress, Challenges and Perspectives

**DOI:** 10.34172/apb.2020.043

**Published:** 2020-05-11

**Authors:** Babak Elyasi Far, Yassin Ahmadi, Ahmad Yari Khosroshahi, Azita Dilmaghani

**Affiliations:** ^1^Drug Applied Research Center, Tabriz University of Medical Sciences, Tabriz, Iran.; ^2^Department of Pharmaceutical Biotechnology, Faculty of Pharmacy, Tabriz University of Medical Sciences, Tabriz, Iran.; ^3^Student Research Committee, Tabriz University of Medical Sciences, Tabriz, Iran.; ^4^Department of Medical Nanotechnology, Faculty of Advanced Medical Science, Tabriz University of Medical Sciences, Tabriz, Iran.

**Keywords:** Microbial, Alpha-amylase, RMS, Stability, Optimization

## Abstract

Alpha-amylase reputes for starch modification by breaking of 1-4 glycosidic bands and is widely applied in different industrial sectors. Microorganisms express unique alpha-amylases with thermostable and halotolerant characteristics dependent on the microorganism’s intrinsic features. Likewise, genetic engineering methods are applied to produce enzymes with higher stability in contrast to wild types. As there are widespread application of α-amylase in industry, optimization methods like RSM are used to improve the production of the enzyme ex vivo. This study aimed to review the latest researches on the production improvement and stability of α-amylase.

## Introduction

Compared to the chemical methods that need harsh conditions such as high pressure and temperature, using of microorganism is considered in many purposes including heavy metal absorption,^1^ gene engeering,^2^ digestion,^3^ production of novel anti-microbs^4^ and particullary for producing of industrial enzymes.^5,6^ Demand on the high-quality productions leads to development of novel methods to improve industrial products such as protease and amylase that are frequently applied in industry and medical science.

There have been identified three types of amylase including α-amylase, β-amylase, and γ-amylase. Alpha-amylase is an industrial enzyme (EC 3.2.1.1.), which cleaves internal alpha 1-4 glycosidic bands of starch and other polysaccharides to produce several products such as glucose and maltose.^7,8^ It belongs to the family of GH13 (most of them), GH57, GH119, and GH126^9^ and is one of the most widely used commercial enzymes.^10^

Most of alpha-amylases are secreted extracellularly, however some intracellular alpha-amylase have been reported. Regarding the outstanding application of α-amylase, there is an urgent need to develop the cost-effective techniques to produce stable and efficient alpha-amylase.^11^ Here we aim to present different methods of the amylase production and how it is possible to improve the efficiency of alpha-amylase.

## Alpha-amylase production in microbial source

A wide range of organisms, including microorganisms such as aquatic bacteria,^12^ fungi, actinomycetes, plants, and animals, can produce alpha-amylase.^13-15^

Regarding the high rate of proliferation and growth, microorganisms are the primary source of alpha-amylase producing a high volume of the enzyme. Also, the genetically manipulated microorganisms are forced to produce alpha-amylase with novel characteristics like thermos-stability.^14,16^ Additionally, the microorganisms produce large quantity of enzyme, which can be simply optimized by various methods such as response surface methodology.^17^ The most widely used microorganisms for the production of alpha-amylase include bacteria, actinomycetes, and fungi.^15^

Several bacteria have been shown are capable of producing a tremendous amount of alpha-amylase for industrial applications, these bacteria include *Bacillus amyloliquefaciens*, *Bacillus licheniformis*, and *Bacillus stearothermophilus*. Some bacteria can produce alpha-amylase in harsh conditions; for instance, some thermophilic bacteria produce alpha-amylase at high temperatures. Most of the starch processing steps, including saccharification, gelatinization, and liquefaction, need high temperature, so the thermostable alpha-amylase is useful to progress the possessing steps in such harsh conditions.^18^ The most common sources of thermostable α-amylase are *Geobacillus bacterium* isolated from Manikaran hot springs. The thermophilic alpha-amylases (BLA) have been shown to have more structural flexibility than mesophilic alpha-amylases (BAA). Although the optimum temperature for this enzyme is 80°C, there is an essential need for amylases resistant to other harsh conditions, especially in the industrial process.^19-21^ The halophilic α-amylase tolerates saline and high-temperature conditions.^14^ Additionally, this enzyme is resistant to organic solvents and keep its activity at low-water conditions. One of the halophilic bacterial sources of alpha-amylase is *Nesterenkonia* sp. strain F, which produces enzymes with catalytic activity even in the organic solvents such as chloroform, benzene, cyclohexane, and toluene.^22^ Due to acidic residues on the surface of halophilic alpha-amylase, this enzyme is stable at low water conditions.^23-25^

Other types of bacteria can remain alive in low temperatures, such as marine bacteria, and produce cold-active enzymes. Cold-active alpha-amylase is widely used in industry for saving energy^26^; for example, in detergent contents, it is not needed to heat water for washing and also cold water help to protect clothes’ color. In the backing process, cold-stable amylase efficiently reduces the time of dough fermentation and improves bread quality. *Pseudoalteromonas* sp. M175 has been isolated from Antarctic sea ice, which is a common source of cold-stable alpha-amylase.^27^ The microorganisms produce cold-active amylases that have a flexible polypeptide chain to make an easier accommodation of substrates at the low-temperature condition. Also, the enzymes contain lipid composition to maintain greater membrane fluidity.^28^

Also, some types of actinomycetes such as *Nocardiopsis aegyptia* can produce cold-adapted enzymes.^15^ Actinomycetes, such as *Streptomyces fragilis* DA7-7 produce thermostable alpha-amylase.^29^

Other microbial sources of alpha-amylase for commercial purposes include *Aspergillus niger*, *Aspergillus awamori*, and *Aspergillus oryzae.*^14^ Due to the extracellular secretion of alpha-amylase that is easily isolatable from the microbial culture medium, fungi can be a good source for α-amylase production in the industry.^30^ Also, other fungi species have been reported to possess some unique features making them suitable for industrial goals; for example, an alpha-amylase produced by *Aspergillus flavus* NSH9 is thermally stable at 50°C.^31^ Table 1 shows different microbial sources for alpha-amylase production.

**Table 1:** Microbial sources for alpha amylase production

**Source**	**Microbial type**	**Feature of alpha-amylase**
*Bacillus stearothermophilu* s	Bacteria	Thermophile alpha-amylase
*Geobacillus bacterium*	Bacteria	Thermophile alpha-amylase
*Nesterenkonia sp*. strain F	Bacteria	Halophilic enzyme
*Bacterium Pseudoalteromonas* sp. M175	Bacteria	Cold-active alpha-amylase
*Nocardiopsis aegypt* ia	*Actinomycetes*	Cold-active alpha-amylase
*Streptomyces fragil* is	*Actinomycetes*	Cold-active alpha-amylase
*Aspergillus nige* r	Fungi	Commercial production
*Aspergillus awamo* ri	Fungi	Commercial production
*Aspergillus oryzae*	Fungi	Commercial production
*Aspergillus flavu* s	Fungi	Thermophile alpha-amylase

## Application of alpha-amylase

Alpha-amylase is currently used in a broad array of industrial applications such as the production of ethanol and high fructose corn syrup, food, textile, paper, and detergent industries.^32-35^ Table 2 shows the most current applications of α-amylase in different industries.

**Table 2:** Industrial applications of α-amylase

**Industry**	**Application**	**Further explanation**	**Microorganism**
Detergent (laundry and dish wash)	Starch stain removal	Detergents containing chemicals don't break down easily in waste-water and cause pollution and eutrophication in the rivers/water bodies so the same may be replaced by enzyme which enhances the detergents ability to remove tough stains and making the detergent environmentally safe. The targeted benefit of enzyme inclusion in detergents is due to much milder conditions; 90% of all liquid detergents contain α-amylase. These enzymes degrade the residues of starchy foods	*Bacillus* or*Aspergillus* (Amylases with activity at lower temperatures, alkaline pH, and oxidative conditions)
Starch liquefaction	Dispersion of insoluble starch granules in aqueous solution and decreasing viscosity followed by partial hydrolysis and	This is the most widespread applications of α-amylases (thermostable) which are used for starch hydrolysis into starch hydrolysates such as glucose and fructose. Because of their high sweetening property, these are used in huge quantities in the beverage industry as sweeteners for soft drinks. Liquefaction process causes reduction in viscosity and partial hydrolysis of starch thus avoided during subsequent cooling.	Bacillus species (thermostable) *amyloliquefaciens ; Bacillus stearothermophilus or Bacillus licheniformis*
Fuel alcohol production	Starch liquefaction and saccharification	Ethanol has been used as fuel a since the early days of the automobile. Ethanol is a significant product of 21st century with its versatile usages and widely consumption across the globe. For the ethanol production, starch is the most used substrate due to its low price and easily available raw material. In this production, starch has to be solubilized and then submitted to two enzymatic steps in order to obtain fermentable sugars. The bioconversion of starch into ethanol involves liquefaction and saccharification, where starch is converted into sugar using an amylolytic microorganism or enzymes such as α-amylase, followed by fermentation, where sugar is converted into ethanol	As yeast *Saccharomyces cerevisiae*
Food industry	Bread softness and volume, flour adjustment; Juice treatment, low calorie beer	Wide applications of α-amylases in food industry include baking, brewing, starch liquefaction as well as a digestive aid (5). They are widely used in baking industry as flavour enhancement and antistaling agent to improve bread quality. α-amylases converts starch to smaller dextrins, which are subsequently fermented by the yeast. improves the taste, crust colour and toasting qualities of the bread; Amylases are also used for the clarification of beer or fruit juices, or for the pretreatment of animal feed to improve the digestibility of fiber	Thermostable maltogenic amylase of *Bacillus stearothermophilus*
Textile industry	De-sizing	Modern production processes for textiles introduce a considerable strain on the warp during weaving. The yarn must, therefore, be prevented from breaking. For this purpose a removable protective layer is applied to the threads. Sizing agents like starch are applied to yarn before fabric production to ensure a fast and secure weaving process. Starch is later removed from the woven fabric in a wet-process in the textile finishing industry. The α-amylases remove selectively the size and do not attack the fibers	*Bacillus* stain
Pulp and paper	De-sizing; Starch-coating, de-inking, drainage improvement	As for textiles, sizing of paper is performed to protect the paper against mechanical damage during processing. It also improves the quality of the finished paper. The size enhances the stiffness and strength in paper. It also improves the erasibilty and is a good coating for the paper.	-

## Alpha-amylase production

### Starch

Starch is known as a carbon source and the main substrate of alpha-amylase, which is comprised of two parts, amylose (25–30%) and amylopectin (70–75%).^36,37^ Amylose contains glucose monomers that are linked to each other via α (1-4) glycosidic bands and its molecular weight spans 1 × 10^5^– 1×10^6^Da. The other polymer is amylopectin, which is polymerized by α(1-4) glycosidic bands and is branched by α(1-6) glycosidic bands with the molecular weight about 1×10^7^- 1×10^9^ Da.^38^

According to the digestibility features, starch is divided into three main groups, including rapidly digestible starch (RDS), slowly digestible starch, and resistant starch (RS).^39^ The most current RDS are used in the foods and gelatinized waxy. RDS in the digestive system is rapidly degraded (20 minutes) into glucose units and so elevates the blood glucose rapidly.^10^

RS is very resistant against digestive mechanisms because of their low glycemic index and these starches are used mainly by bacteria in the colon to generate short-chain fatty acids, which is essential for human health. RS consists of five different types, including encapsulated starch, resistant granules, retrograded amylose, chemically modified starch, and amylose-lipid complex.^37^

### Production method

Isolated microbial strains used in starch processing must be able to produce the enzyme on the industrial scale. To produce alpha-amylase on the industrial scale, submerged fermentation (SMF), and solid state fermentation (SSF) are frequently utilized. SMF is used to produce bio-products from broth medium such as molasses. This method requires remarkable moisture that is crucial for the growth of microorganisms (mostly bacteria) in the medium to produce alpha-amylase.^14^ This high moisture also provides easily applicable processes for sterilization, production, purification, controllable temperature, nutrient, pH, and etc; for instance, amylase production as a microbial source using *Bacillus* sp. ^40^

SSF is used for the production of alpha-amylase from easily recycled waste materials such as paper. This method needs low moisture, which can be regarded as an advantage. Further advantages of SSF include more straightforward equipment, more production, and less effluent production. However, SSF is slower than SMF in utilizing of substrates by microorganisms. Therefore, SSF is the most common method for the production of alpha-amylase.^14^ *Bacillus thuringiensis* and *Bacillus cereus* have been frequently used in co-culture condition to produce alpha-amylase through SSF.^41^

### Enzyme activity assay

**Figure 1:**
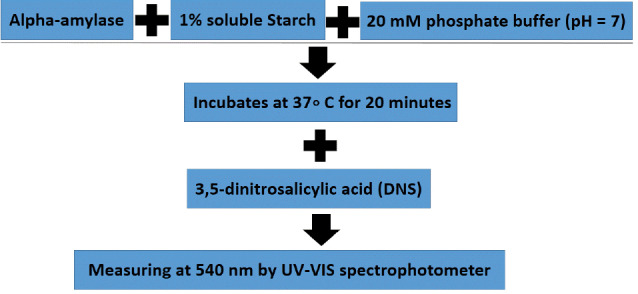
Assaying of alpha-amylase activity by DNS method.

The digestive activity of alpha-amylases is measured via several colorimetric methods, including dinitrosalicylic acid method (DNS), Nelson–Somogyi, and Iodine method.^42^ DNS is an alkaline reagent that attaches to the reducing sugars and then color changes can be detected by UV absorbance at 540 nm^43^ (Figure 1). Amylases, xyloglucanases, pectinases, and β-mannanases are assessed by DNS method. A Drawback of this method is the lack of information on its stoichiometric properties with oligosaccharides^44^ (Figure 1).

**Figure 2:**
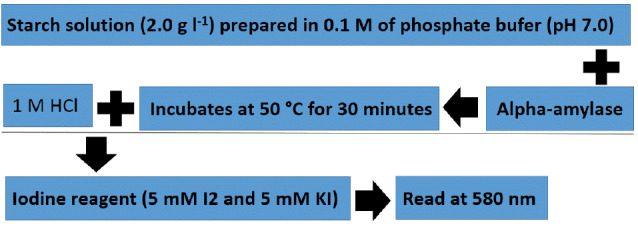
Assaying of alpha-amylase activity by iodine method.

**Figure 3:**
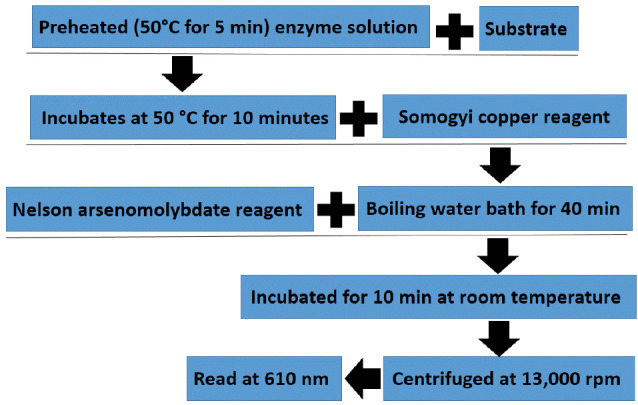
Assaying of alpha-amylase activity by Nelson-Somogyi method.

In iodine method, Lugol interacts with starch and forms complexes and alpha-amylase degrades starches and reduces UV absorbance at 580 nm.^31^ Figure 2 shows measurement of the alpha-amylase activity by iodine method. Also, While Nelson – Somogyi method is used for measuring of α-amylase activity. This method is 10 times more sensitive than DNS method.^44^ DNS method process is described in Figure 3.

### Medium composition factors 

The condition of the culture medium must be regarded for enzyme production. Medium composition and physical conditions can directly affect the production of alpha-amylase. Different factors have been shown to affect enzyme production, here these factors are presented.

#### Carbon source

Some of the most known substrates as a carbon source for microorganisms to produce alpha-amylase include maltose, glucose, and sucrose. A study on *Aspergillus oryzae* S2 showed that the proper concentration of starch (10%) are the best carbon source to produce alpha-amylase.^45^ *Penicillium notatum* IBGE 03 is another fungus that is used for alpha-amylase production; this fungus uses molasses as its favorable carbon source.^42^ *Bacillus subtilis* as another microorganism is used for the production of alpha-amylase in SSF and it needs glucose for optimized production of alpha-amylase is 0.02 g/g.^46^ Another study on *Bacillus* family showed that the glucose concentration for optimum production of alpha-amylase by *Bacillus amyloliquefaciens* was 10.50 g/L.^47^

#### pH optimization

pH plays a significant role in the production and secretion of alpha-amylase. Microorganisms need an appropriate growth condition for production of amylase, for example, most fungi grow in the light acidic condition; however, bacteria need a neutral pH (around pH 7).^48^

*Kluyveromyces marxianus* IF0 0288 at pH 6.13 produces the highest quantity of alpha-amylase.^49^ The optimized pH for the production of alpha-amylase by *Penicillium notatum* IBGE 03 has been reported to be 5.5.^42^ Also, *Bacillus sp.* MB6 produces a remarkable quantity of alpha-amylase at pH 6.^50^

#### Nitrogen source

As nitrogen contents in culture medium have a significant role in the growth of microorganisms. Different nitrogen sources have been widely studied for the optimization of alpha-amylase production, including the organics such as yeast extract, soybean, and peptone, which are the most applicable nitrogen sources in culture medium; other sources are the inorganics, such as ammonium hydrogen phosphate, ammonium sulfate, and ammonium chloride.^14^

*Bacillus amyloliquefaciens* KCP2 produces a significant amount of alpha-amylase using ammonium sulfate (0.2 g*)* as an inorganic nitrogen source under SSF.^51^ Also, *Bacillus amyloliquefaciens* has been shown to use yeast extract (2 g/L), as an organic nitrogen source to produce alpha-amylase.^52^ A study on *Penicillium notatum* IBGE 03 used corn steep as the organic nitrogen source to optimize alpha-amylase production.^42^ Soybean has been used as a source of nitrogen by *Aspergillus oryzae* CBS 819.72, which produced alpha-amylase on optimized condition.^36^

### Metal ions

Ca^2+^ ions due to presence in alpha-amylase structure play an important role in alpha-amylase production; In most culture media, calcium chloride (CaCl_2_) is added to produce alpha-amylase.^48^

*Bacillus amyloliquefaciens* has been shown to use CaCl_2_ (0.0275 M) as a crucial factor to produce alpha-amylase.^53^ Also, further study by Zhao et al showed that CaCl_2_ (2 g/L) plays an important role in the production of alpha-amylase by *Bacillus amyloliquefaciens.*^52^ *Penicillium* sp*.,* another microbial source is remarkably dependent on CaCl_2_ to produce high levels of alpha-amylase.^54^

### Enzyme optimization methods 

Hydrolytic enzymes contribute to global business so there is an essential need for optimization of these enzymes. Some methods have been developed for optimization of these enzymes, including response surface methodology^55^ and Taguchi methods.

#### Response surface methodology

Due to the vital role for the optimization of factors and developing a novel experiment, RSM is considered as an important part of the experimental design.^52^ RSM is comprised of statistical and mathematical techniques consisting of two methods for optimization, including Box–Behnken designs and Central Composite Design (CCD).^56^ Minitab^®^ 16.1.0 is the most commonly used software for the optimization of amylase production by *Enterococcus faecium* DMF78.^57^ On the other hand, design expert (version 8.0) is frequently utilized to improve the alpha-amylase production from thermostable and alklophilic alpha-amylase from *Bacillus amyloliquefaciens* KCP2.^51^

**Figure 4:**
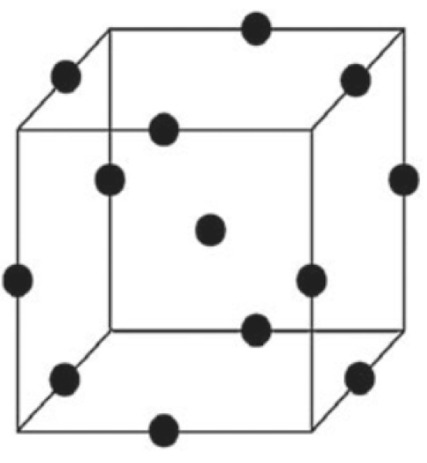
Schemes of Box–Behnken design.

Box–Behnken designs:The Box–Behnken designs have been used for RSM experiment to enhance alpha-amylase production from *Aspergillus oryzae* S2, *Aspergillus oryzae* CBS 819.72, *Bacillus laterosporus*, and *Bacillus amyloliquefaciens*^36,45,52,58^ (Figure 4).

**Figure 5:**
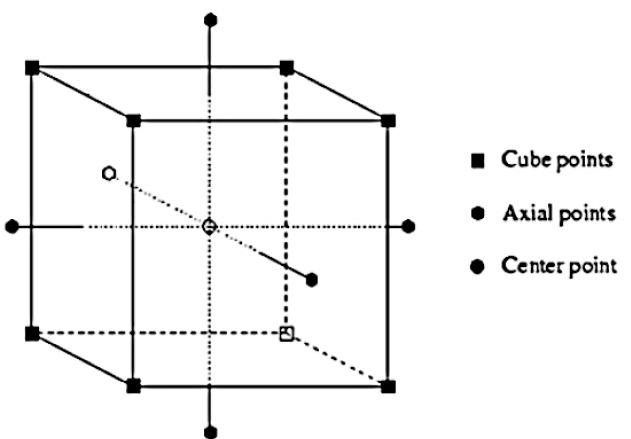
Schemes of Central Composite Design (CCD).

CCD: Figure 5 shows CCD.*Bacillus amyloliquefaciens , Streptomyces erumpens* MTCC 731, *Bacillus amyloliquefaciens* KCP2, *and Aspergillus oryzae* S2 synthesize alpha-amylase by CCD method.^51,59-61^

### Purification

Purification of the produced enzyme may include the main part of the enzyme production cost, in particular when there is a need for stringent purification. There are different methods of enzyme purification; however, selecting the final method is dependent on the market, cost, final quality, and available technology.^62^

Mostly, after primary isolation via precipitation or membrane separations, enzymes are purified by chromatographic methods.^11,63,64^ Regarding the needs for large-scale production of enzyme, the purification methods have been improved to provide higher efficiency, cost-effectiveness, and faster purification in less processing steps.^65^ Table 3 shows several purification methods for amylase in bacteria.

**Table 3:** Purification methods for alpha amylase

**Microorganism**	**Method of purification**	**Yield %**
Thermostable, thermophilic, alkaline and chelator resistant amylase from an alkaliphilic *Bacillus sp*. Isolate ANT-6	Partial purification by ethanol precipitation	-
Marine *Vibrio sp*.	Substitute affinity method by insoluble corn stratch	78
*Bacillus subtilis*	High-speed counter-current chromatography	73
*Bacillus amyloliquefaciens*	Primary precipitation by ammonium sulphate then final purification by ion exchange chromatography	-
Thermostable α -amylase from *Bacillus sp*. PS-7	Partial purification with ammonium sulphate and further puriﬁcation by Sephadex G-75 gelﬁltration followed by phenyl agarose 4XL hydrophobic interaction chromatography.	-
*Bacillus amyloliquefaciens*	Afﬁnity precipitation by alginate as the afﬁnity matrix.	96
-	Affinity adsorption chromatography	95
-	Expanded bed chromatography	69

### Stability of alpha-amylase

Industrial enzymes should be stable in harsh conditions. One of the industrial enzymes is alpha-amylase, which needs to be stable against high temperatures, pH, oxidation, and other conditions that are inevitable during starch hydrolysis. So far, several methods have been utilized to achieve a stable amylase; the first, isolation and screening enzyme for extremophile microorganisms such as thermophile bacteria and genetically manipulating of other microorganisms; the second, improving enzyme stability via protein engineering, enzyme immobilization and adding of additives.^66^

Enzyme recycling maybe regarded as a strategy to save the enzyme. The limitation of recycling of the enzyme was solved by immobilization method.^67^ Also, immobilization provides higher stability, and continuous operation, and it is suitable for several various engineering designs and improves control of reaction.^68^ Several methods have been used for Immobilization, including entrapment, crosslinking, covalent attachment, encapsulation, and adsorption.^66^

Due to fungal alpha-amylases recognized as safe status, the alpha-amylases are more preferred in the starch industry but it needs to be more stable and cost-effective. A study by He et al showed increased stability and longer service life of fungal alpha-amylases (AmyA1) via immobilization on magnetic nanoparticles.^69^

Another method for enhancing enzyme stability is protein engineering, which is classified into two categories; the site-directed mutagenesis and the random mutagenesis. Improving stability by random mutagenesis within the whole length of a gene uses several methods such as UV irradiation, DNA shuffling, chemical mutation, and error-prone PCR.^66^ Site-directed mutagenesis is an approach to produce new biocatalyst with a higher stability, activity, expiration, and solubility compared with wild protein. Appropriate amino acid residues are selected and manipulated, is the leading characteristic of the site-directed mutagenesis.^70^

A useful effect of mutagenesis on the stability of amylase was shown by Wang et al. Most of the Alpha-amylases are active in natural pH. Due to acidic pH of native starch slurry (3.2-45) deactivates the enzyme during starch processing, starch slurry must be adjusted to natural pH. A recent report on alpha-amylase production by *Bacillus subtilis* showed that the mutated alpha-amylase (in the active site) is more active in acidic condition.^71^

Another method for improving the alpha-amylase stability is chemical modification.^72^ In the starch industry, high temperatures are required for synthesis of the product, which inactivates alpha-amylase. Siddiqui et al reported that the modification of carboxyl groups of TAA by L-arginine methyl ester dihydrochloride may improve the hydrolytic activity of alpha-amylase at 60°C.^73^

Water is crucial for alpha-amylase activity; on the other hand, water can lead to denaturation of the enzyme. some stabilizing agents such as sugars or polyols can be added to alpha-amylase in order to change water structure and improve hydrophobic interaction strength in the enzyme structure.^66,74^ Samborska et al reported that sucrose, trehalose, and polyols can have a remarkable stabilizing effect on alpha-amylase at thermophilic condition.^75^

## Conclusion

The production of alpha-amylase at a large scale are crucial for industrial goals. Therefore, finding microorganisms with such potential to produce a high quantity of amylase is an important goal of the scientists. Some microorganisms from special environments such as saline soil can be used to achieve such goal. Also according to the microorganism sources, some types of amylases can be used to save energy via the development of the cold-active alpha-amylase. Also, protein engineering methods can be applied to produce enzymes with remarkable stability.

## Ethical Issues

Not applicable.

## Conflict of Interest

Authors declare that there is no conflict of interest.

## Acknowledgments

This project (Ph.D.thesis) is financially supported by Tabriz University of Medical Sciences, Tabriz, Iran (Grant Number: 58056).
